# Extract of *Cornus officinalis* Protects Keratinocytes from Particulate Matter-induced Oxidative Stress

**DOI:** 10.7150/ijms.36476

**Published:** 2020-01-01

**Authors:** Pincha Devage Sameera Madushan Fernando, Mei Jing Piao, Ao Xuan Zhen, Mee Jung Ahn, Joo Mi Yi, Yung Hyun Choi, Jin Won Hyun

**Affiliations:** 1 Department of Biochemistry, College of Medicine, Jeju National University, Jeju 63243, Republic of Korea.; 2 Laboratory of Veterinary Anatomy, College of Veterinary Medicine, Jeju National University, Jeju 63243, Republic of Korea.; 3 Department of Microbiology and Immunology, College of Medicine, Inje University, Busan 47392, Republic of Korea.; 4 Department of Biochemistry, College of Oriental Medicine, Dongeui University, Busan 47340, Republic of Korea.

**Keywords:** ethanol extract of *Cornus officinalis* fruits (EECF), particulate matter 2.5 (PM_2.5_), human HaCaT keratinocytes, oxidative stress

## Abstract

The skin is one of the large organs in the human body and the most exposed to outdoor contaminants such as particulate matter < 2.5 µm (PM_2.5_). Recently, we reported that PM_2.5_ induced cellular macromolecule disruption of lipids, proteins, and DNA, via reactive oxygen species, eventually causing cellular apoptosis of human keratinocytes. In this study, the ethanol extract of *Cornus officinalis* fruit (EECF) showed anti-oxidant effect against PM_2.5_-induced cellular oxidative stress. EECF protected cells against PM_2.5_-induced DNA damage, lipid peroxidation, and protein carbonylation. PM_2.5_ up-regulated intracellular and mitochondrial Ca^2+^ levels excessively, which led to mitochondrial depolarization and cellular apoptosis. However, EECF suppressed the PM_2.5_-induced excessive Ca^2+^ accumulation and inhibited apoptosis. The data confirmed that EECF greatly protected human HaCaT keratinocytes from PM_2.5_-induced oxidative stress.

## Introduction

Particulate matter (PM) is an air pollutant with harmful effects on the human skin that contribute to conditions such as skin cancers, alopecia, and skin aging 1,2. In particular, the harmful effects of PM depend on the composition of deleterious contents such as heavy metals (Cu, Mn, Ni, Pb, and Ti) and polycyclic aromatic hydrocarbons 3. PM < 2.5 µm (PM_2.5_) is considered fine PM and its detrimental effects on the human skin are mediated by the generation of excessive intracellular reactive oxygen species (ROS), which creates oxidative stress 4-6. PM_2.5_-mediated excessive ROS generation could elicit lipid peroxidation, DNA damage, apoptotic protein expression, and mitochondria-dependent apoptosis, which eventually results in skin irritation and damage 7.

There are more than 65 species classified under the genus *Cornus* (family Cornaceae), but only two species, *Cornus mas* and *Cornus officinalis*, have been reported as medicinal plants used in traditional medicine 8. These plants are mainly distributed in eastern Asia including Korea, Japan, and China. *C. officinalis* is commonly known as cornel dogwood or Asiatic dogwood 9. *C. officinalis* grows up to 4-10 m high, has papery leaves that are 5.5-10 cm long, its flowers consist of four petals with a yellow lanceolate tongue that is 3.3 mm long 10. *C. officinalis* fruit has been used to treat high blood pressure, kidney deficiency, dizziness, spermatorrhea, and waist and knee pain since ancient times 10,11. Most related pharmacological studies have revealed that the ethanol extract of *C. officinalis* fruit (EECF) possesses anti-hyperglycemia, anti-aging, immune-regulatory, and renal and neuro-protective effects 12. In addition, the neuro-protective, antioxidant, anti-inflammatory, cardiovascular, and anti-diabetic effects of the EECF have been revealed 13. Furthermore, *C. officinalis* fruit contain high amounts of volatile compounds, organic acids, carbohydrates, tannins, and iridoids. Particularly, iridoid glycosides are one of the active ingredients in the *C. officinalis* fruit 14. However, there are few reports of the cytoprotective effect of EECF against PM_2.5_-induced oxidative stress in human keratinocytes. Therefore, this study was conducted to investigate the potential of EECF to cure the PM_2.5_-induced cell damage.

## Materials and methods

### Reagents and chemicals

The dried fruit of *C. officinalis* collected from an area around the city of Gurye (Jeollanam-do Province, Republic of Korea), were provided by Gurye Sansuyu Farming Association Corporation. For the preparation of EECF, the dried fruit (20 g) were cut into small pieces and extracted three times with 400 mL 70% ethanol at 4°C for 3 h. After filtering, the filtrate was concentrated using a vacuum rotary evaporator (EYELA SB-1000, Tokyo Rikakikai Co. Ltd., Tokyo, Japan). The residue was then freeze-dried using a freeze dryer and stored at -80°C. The powder (EECF) was dissolved in dimethyl sulfoxide (DMSO, Sigma‑Aldrich Chemical Co., St. Louis, MO, USA) to obtain a final concentration of 100 mg/mL (extract stock solution), and was stored at 4°C. The stock solution was diluted with culture medium to the desired concentrations prior to use. EECF was dissolved in DMSO. Diesel PM_2.5_ (NIST SRM 1650b, PM_2.5_) was purchased from Sigma‑Aldrich Chemical Co. and was dissolved in DMSO to prepare the stock solution (25 mg/mL). To avoid agglomeration of the suspended PM_2.5_, the solution was sonicated for 30 min 15.

### Cell culture

The human HaCaT keratinocytes (Cell Line Service, Heidelberg, Germany) were cultured in Dulbecco's modified Eagle's medium (DMEM, Life Technologies Corporation, Staley Rd, Grand Island, USA). The medium was supplemented with antibiotic solution consisting of 100 units/mL penicillin, 100 µg/mL streptomycin, and 0.25 µg/mL amphotericin B (Gibco, Life Technologies Co., Grand Island, NY, USA). In addition, the medium was supplemented with 10% fetal bovine serum. The cultured cells were incubated in a 100% humidified atmosphere at 37°C with 5% CO_2._

### Cell viability

The cytotoxicity of the EECF on HaCaT cells was measured using the 3-(4,5-dimethylthiazol-2-yl)-2,5-diphenyl tetrazolium bromide (MTT) assay. Cells were cultured in a 96-well plate at a density of 1.0 × 10^5^ cells per well and specific wells were separately treated with EECF at final concentrations of 25, 50, 100, 200, 300, 400, and 500 µg/mL. The MTT stock solution (2 mg/mL) was incubated with the cells for 4 h until formazan crystals were formed. The crystals were then dissolved in DMSO and the absorbance of the reaction solution was detected using a multi-well spectrophotometer at a wavelength of 540 nm.

### DPPH radical detection

EECF (25, 50, 100, 200, 300, 400, and 500 µg/mL) was mixed with 0.15 mM 2,2-diphenyl-1-picrylhydrazyl (DPPH), shaken gently, and kept in the dark for 3 h. The residual DPPH was determined at 520 nm using a spectrophotometer.

### ROS detection

Cells were seeded in 96 well palate at a 1.5 × 10^5^ cell density and intracellular ROS levels those generated via 1 mM hydrogen peroxide (H_2_O_2_), were measured using the 2',7'-dichlorofluorescein diacetate (DCF-DA, Sigma‑Aldrich) assay. Cells were seeded on chamber slides at a 1.5 × 10^5^ cell density and incubated with PM_2.5_ (50 µg/mL) for 1 h. The cells were stained with DCF-DA for 30 min and the fluorescence emission was detected using confocal microscopy (Carl Zeiss, Oberkochen, Germany).

### Detection of superoxide anion

Superoxide anion was generated though the reaction between 5,5-dimethylpyrroline-N-oxide (DMPO) and the xanthine/xanthine oxidase system. The generated DMPO/·OOH adduct was detected using electron spin resonance (ESR). Then, 20 µL xanthine oxidase (0.25 U/mL) was mixed with 20 µL each of xanthine (10 mM), EECF (200 µg/mL), and 3 M DMPO, and after 2.5 min, the ESR signaling was measured. ESR spectrophotometer settings were set as follows: power, 1.00 mW; central magnetic field, 336.8 mT; frequency, 9.4380 GHz; amplitude, 600; modulation width, 0.2 mT; sweep width, 10 mT; sweep time, 30 sec; gain, 500; time constant, 0.03 sec; temperature, 25°C 16.

### Lipid peroxidation assay

A four-well chamber slide was used to plate the cells in the presence of 200 µg/mL EECF, followed by exposure to PM_2.5_ (50 µg/mL) for 24 h and staining with diphenyl-1-pyrenylphosphine (DPPP) for 30 min in the dark. Images were analyzed using a confocal microscope 15.

### Protein carbonylation assay

Cells were incubated with 200 µg/mL EECF for 1 h and treated with PM_2.5_ (50 µg/mL) for 24 h. Protein oxidation was assessed using an OxiSelect^TM^ protein carbonyl enzyme-linked immunosorbent assay kit (Cell Biolabs, San Diego, CA, USA) according to the manufacturer's instructions.

### Single-cell gel electrophoresis

Cells were seeded in the medium with 200 µg/mL EECF in a 1 mL micro tube for 30 min and treated with PM_2.5_ (50 µg/mL) for another 30 min. After coating with 110 µL 0.7% low-melting agarose, the cells were immersed in lysis buffer (2.5 M NaCl, 100 mM Na_2_EDTA, 10 mM Tris, 1% N-lauroylsarcossinate) for 1 h at 4°C. An electrical field (300 mA, 25 V) was used for electrophoresis. Slides were stained with 40 µL ethidium bromide (10 µg/mL) and analyzed using the comet 5.5 image analyzer (Andor Technology, Belfast, UK). The percentage total fluorescence and tail lengths were recorded (50 cells per slide).

### Detection of 8-oxoguanine (8-oxoG) expression

ROS-induced DNA damage was assessed using the avidin-tetramethylrhodamine isothiocyanate (TRITC, 1:200) conjugate (Sigma-Aldrich) assay, based on fluorescence-binding activity. Initially, the cells were fixed on chamber slides at a 1.5 × 10^5^ cell density and images of fluorescence-visualized cells were captured using a confocal microscope 17.

### Detection of Ca^2+^ level

Treated cells were loaded with 10 µM fluoro-4-acetoxymethyl ester (Fluo-4-AM) or rhod-2-acetoxymethyl ester (Rhod-2-AM, Molecular Probes) for 30 min to detect intracellular Ca^2+^ level and mitochondrial Ca^2+^, respectively. Fluorescence was measured using confocal microscopy 18.

### Mitochondrial membrane potential (Δψ_m_) analysis

Cells were seeded on chamber slides at a density of 1.5 × 10^5^ cells. After treatment with EECF (200 µg/mL), cells were exposed to PM_2.5_ (50 µg/mL) for 24 h, stained with 5 μM 5,5′,6,6′-tetrachloro-1,1′,3,3′-tetraethylbenzimidazolylcarbocyanine iodide (JC-1, Invitrogen, Carlsbad, CA, USA), and analyzed using confocal microscopy.

### Western blotting

Harvested cells were lysed using 150 μL of protein lysis buffer and the collected cell lysates were centrifuged at 13,000 rpm for 5 min. The resulting suspensions were collected and protein levels were analyzed as previously described 19. Aliquots were electrophoresed using 12% sodium dodecyl sulfate-polyacrylamide gel electrophoresis. Then, the separated proteins were transferred onto the nitrocellulose membranes, which were sequentially incubated with the appropriate primary and secondary antibodies. Protein bands were detected using the Amersham ECL western blotting detection reagents and analysis system (GE healthcare, Amersham place, UK).

### Hoechst 33342 staining

Cells were treated with 200 µg/mL EECF for 1 h, followed by PM_2.5_ (50 µg/mL) for 18 h. The cells were stained with Hoechst 33342 (20 µM) and DNA-specific fluorescence was visualized using a fluorescence microscope. Nuclear condensation levels were evaluated and quantified for the apoptotic cells.

### Statistical analysis

All experiments were performed in triplicate. Data are presented as the means ± standard error and were analyzed using the Sigma Stat 3.5 version software (Systat Software Inc., San Jose, CA, USA) using Tukey's test and analysis of variance (ANOVA). A *P* < 0.05 was considered statistically significant.

## Results

### EECF reduced ROS generation

**Figure 1:**
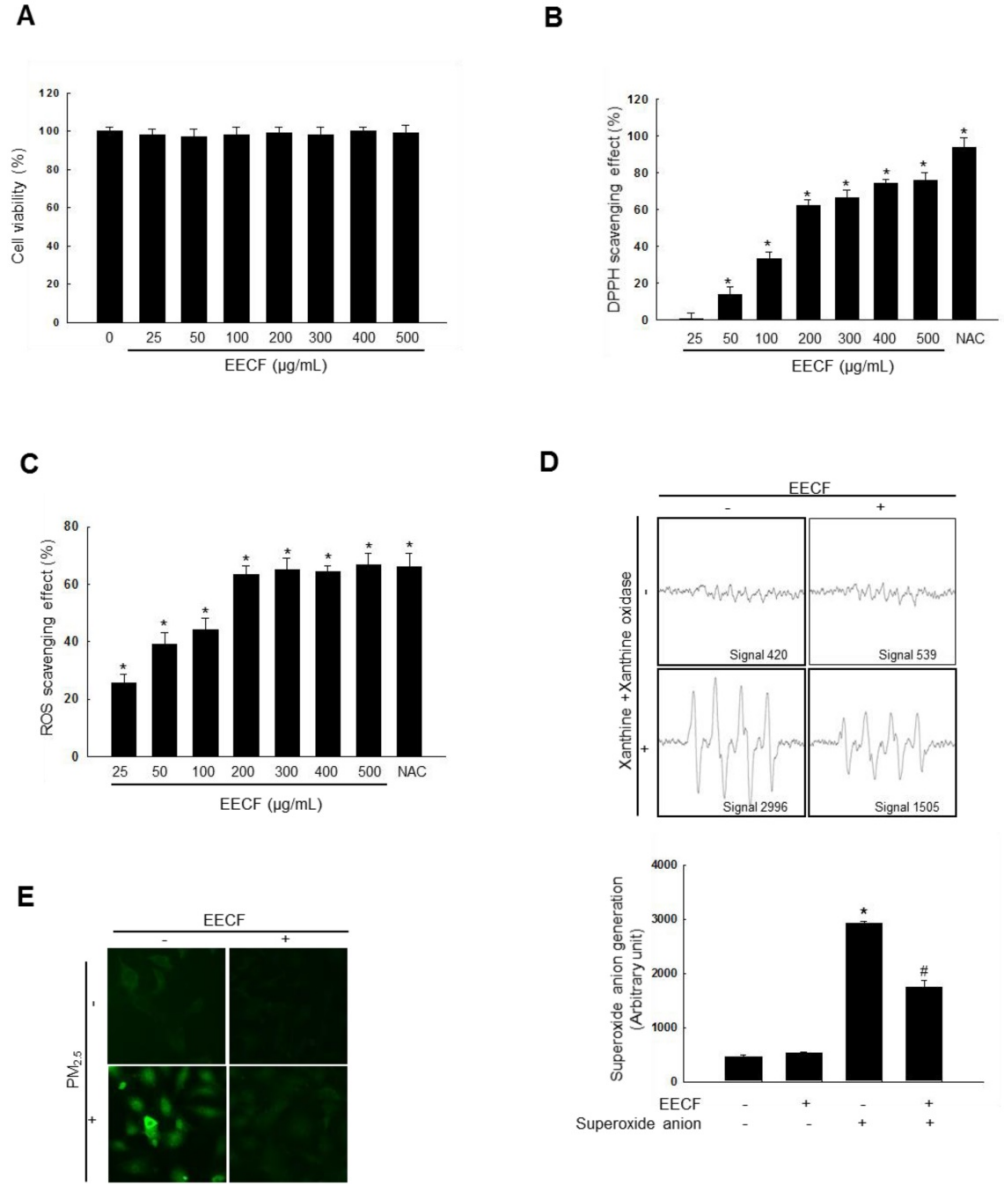
Ethanol extract of *C. officinalis* fruit** (**EECF) reduced ROS generation. (A) MTT assay was used to assess cell viability of EECF (0, 25, 50, 100, 200, 300, 400, and 500 µg/mL)-treated HaCaT cells for 24 h. (B) Radical-scavenging effects of EECF were investigated using DPPH assay. ^*^p < 0.05 compared with control. (C) Intracellular ROS level that generated by H_2_O_2_ (1mM), was detected using spectrophotometer after DCF-DA staining. NAC is the positive control. ^*^p < 0.05 compared with control. (D) Superoxide anion reducing ability of 200 µg/mL EECF was investigated using xanthine/xanthine oxidase system. ^*^p < 0.05 and ^#^*p* < 0.05, compared with control and superoxide anion-treated group, respectively. (E) Effect of EECF on PM_2.5_-induced intracellular ROS generation was assessed using DCF-DA staining by confocal microscopy.

Before commencing the experiment, we sought to determine if EECF had any cytotoxicity on human HaCaT keratinocytes using the MTT assay with different EECF concentrations (0, 25, 50, 100, 200, 300, 400, and 500 µg/mL, Figure 1A). The results confirmed that EECF was not cytotoxic against HaCaT cells at any of the tested concentrations. EECF showed DPPH radical scavenging activity at all the tested concentrations compared with N-acetylcysteine (NAC), a well-known antioxidant (Figure 1B). Next, the ROS scavenging ability of EECF was tested, and concentrations of 25-200 µg/mL showed rapidly increasing ROS (generated via 1 mM H_2_O_2,_ respectively) scavenging activity and, therefore, 200 µg/mL was selected as the optimal concentration for further experiments (Figure 1C). To assess the ability of EECF (200 µg/mL) to scavenge superoxide anion, ESR spectrometry was performed. Superoxide anions produced by the xanthine/xanthine oxidase system were reduced by EECF, as shown in Figure 1D. The generated signal of 2,996 in the control was reduced to 1,505 in the presence of EECF. Intracellular ROS generation assessed using the DCF-DA assay revealed that 200 µg/mL EECF ameliorated the green color intensity caused by the PM_2.5_, which was visualized using confocal microscopy (Figure 1E).

### EECF significantly attenuated PM_2.5_-induced lipid peroxidation, protein carbonylation, and DNA damage

**Figure 2:**
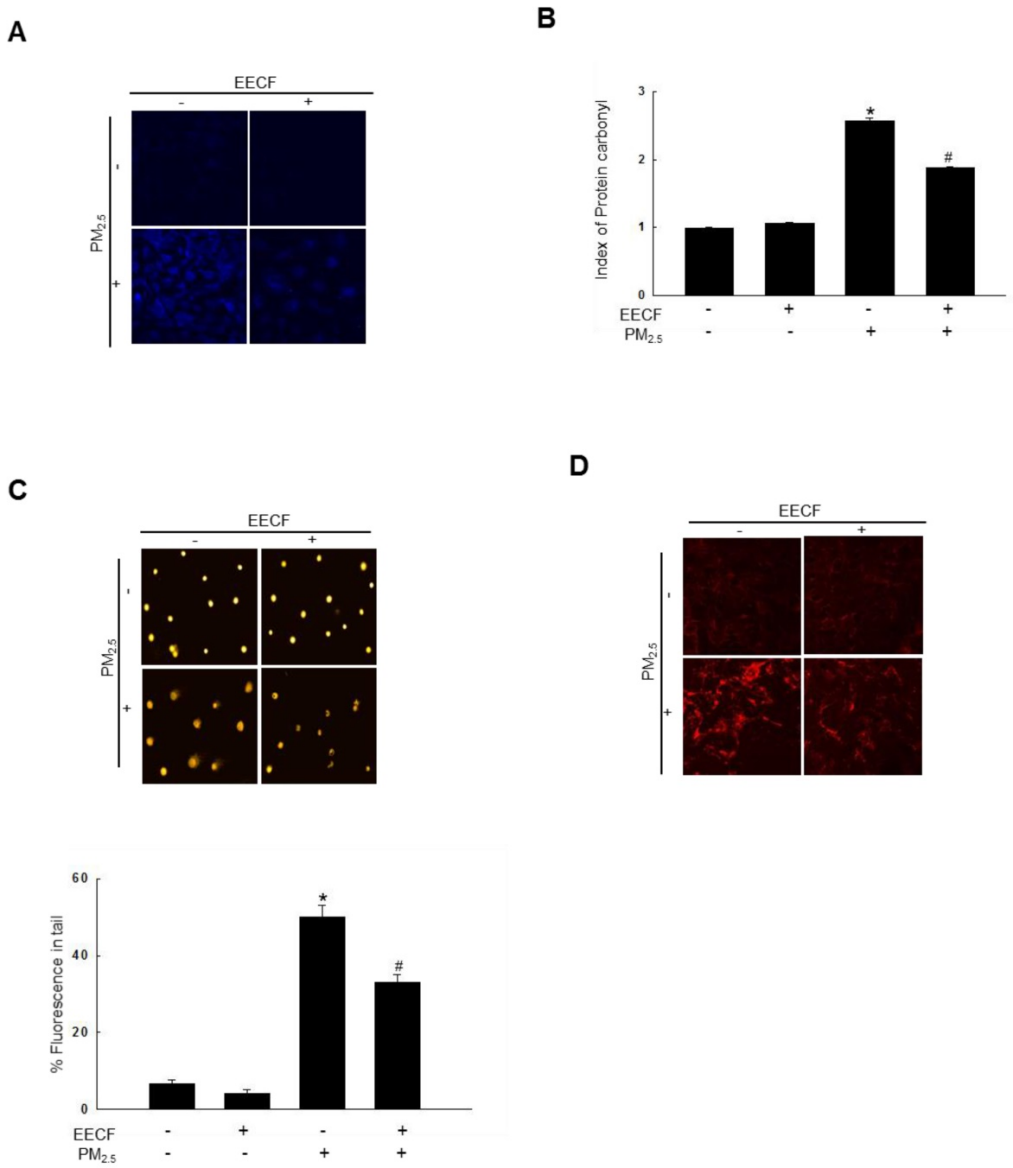
Ethanol extract of *C. officinalis* fruit** (**EECF) protected cells against PM_2.5-_induced lipid peroxidation, protein carbonylation, and DNA damage. (A) EECF effect on PM_2.5_-induced lipid peroxidation was assessed using confocal microscopy after DPPP staining. (B) Protein oxidation was assessed by measuring carbonyl formation. ^*^p < 0.05 and ^#^*p* < 0.05, compared to control and PM_2.5_-treated group, respectively. (C) DNA damage was assessed using comet assay. ^*^p < 0.05 and ^#^*p* < 0.05, compared to control and PM_2.5_-treated group, respectively. (D) Avidin-TRITC conjugate was examined to evaluate DNA oxidative adducts of 8-oxoG using confocal microscopy.

The lipid peroxidation amount was assessed by visualizing the fluorescent intensity of oxidized DPPP, which is an indicator of lipid peroxidation. The DPPP oxidase intensity was higher in PM_2.5_-treated cells than it was in control cells. Pretreatment with EECF significantly reduced the florescent intensity of PM_2.5_-containing cells (Figure 2A). The results indicated that EECF treatment has the potential to reduce ROS generation and further confirmed the ROS scavenging properties. Then, protein carbonylation was measured. Carbonyl groups are formed during the process of protein oxidation 16. PM_2.5_ significantly increased the expression of carbonyl moieties, whereas EECF-pretreated cells exhibited notably reduced formation of protein carbonyl when they were exposed to PM_2.5_ (Figure 2B). Furthermore, PM_2.5_-induced DNA damage was monitored using a comet assay. As shown in Figure 2C, treatment with the PM_2.5_ distinctly elongated the comet tail and increased the damaged DNA around the nuclei. Pre-treatment of HaCaT cells with EECF before exposure to PM_2.5_ obviously reduced the level of damaged DNA in comet tails. Finally, the level of 8-oxoG was analyzed using confocal microscopy, and PM_2.5_-treated cells showed the highest 8-oxoG level. PM_2.5_ exposure caused severe DNA lesions in cells, which were revealed by avidin-TRITC binding. Furthermore, EECF was shown to attenuate the PM_2.5_-induced DNA lesions (Figure 2D).

### EECF attenuated PM_ 2.5_-induced mitochondrial stress

**Figure 3:**
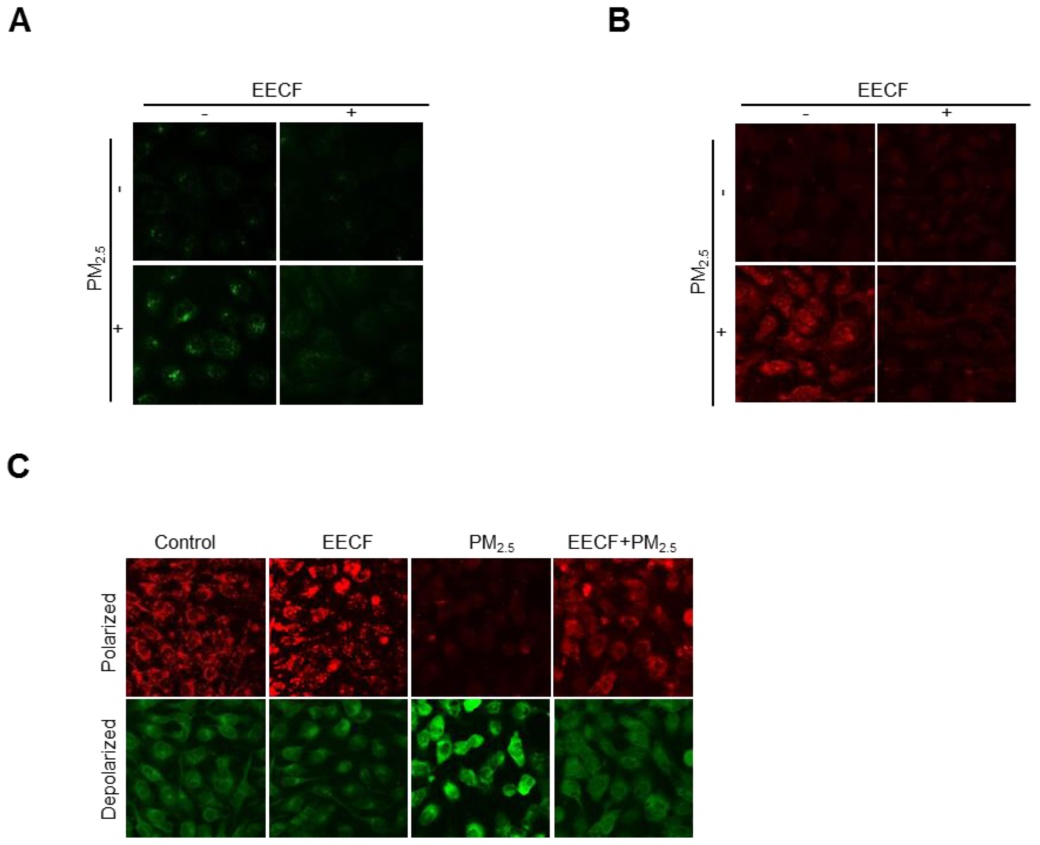
Ethanol extract of *C. officinalis* fruit** (**EECF) attenuated PM_2.5_-induced mitochondrial stress. (A) Effect of EECF on intracellular Ca^2+^ was assessed using confocal microscopy after Fluo-4-AM staining. (B) EECF effect on the mitochondrial Ca^2+^ level was assessed using confocal microscopy after Rhod-2-AM staining. (C) Mitochondrial membrane potential (Δψ_m_) was detected using JC-1 staining.

Initially, we hypothesized that the oxidative ability of PM_2.5_ was mediated by mitochondrial stress. Therefore, intracellular Ca^2+^ level were assessed, because previous studies have reported that disruption of Ca^2+^ homeostasis generates mitochondrial stress 15. Cells were stained with Fluo-4-AM dye, and confocal microscopy analysis revealed that Ca^2+^ fluorescence was much higher in the PM_2.5_-treated group than in the other cells. Pretreatment with EECF obviously reduced the intracellular Ca^2+^ level of PM_2.5_-treated cells (Figure 3A). The mitochondrial Ca^2+^ level was assessed by staining cells with Rhod-2-AM dye and the confocal microscopy analysis revealed that treatment with EECF notably reduced the level (Figure 3B). The Δψ_m_ was assessed using JC-1 dye, where red and green fluorescence indicated the polarized and depolarized state of the mitochondria, respectively 20. The results indicated that mitochondrial depolarization was enhanced by PM_2.5_ but was notably reduced by EECF, as shown in the confocal microscopy analysis (Figure 3C).

### PM_2.5_-induced cell apoptosis was attenuated by EECF

**Figure 4:**
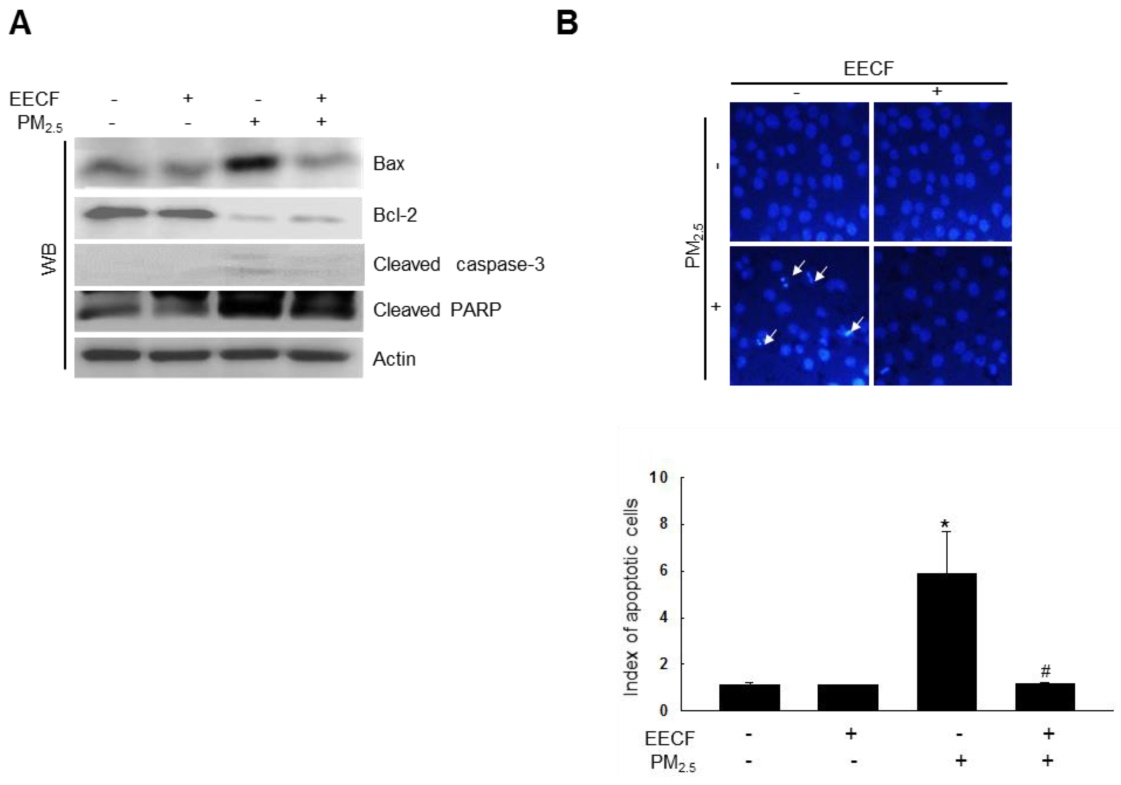
Ethanol extract of *C. officinalis* fruit (EECF) attenuated the PM_2.5_-induced cellular apoptosis. (A) Cell lysates were analyzed for Bax, Bcl-2, cleaved caspase-3, and cleaved PARP protein expression using western blot (WB). (B) Cells were stained with Hoechst 33342 dye and apoptotic cells were observed and quantified. The arrow indicates apoptotic cells. ^*^p < 0.05 and ^#^*p* < 0.05, compared to control and PM_2.5_-treated group, respectively.

PM_2.5_ enhanced the expression of cleaved caspase-3, cleaved poly-ADP ribose polymerase (PARP) and B-cell lymphoma-2-associated X protein (Bax) and decreased the expression of B-cell lymphoma-2 (Bcl-2) (Figure 4A). This finding suggests that caspase-3 was likely involved in the observed cell apoptosis. However, pretreatment with EECF attenuated the cell apoptosis, and the cell nuclei stained with Hoechst 33342 were analyzed using microscopy, which showed significant nuclear condensation in PM_2.5_-treated cells. Cells pretreated with EECF were observed to be normal (Figure 4B).

## Discussion

The skin is the largest organ in the body and it protects the body by acting as a barrier to the external environment 21-23. PM_2.5_ is considered an air pollutant, which has harmful effects on the skin, such as skin aging and inflammatory skin diseases, mediated by the generation of intracellular ROS 24,25. One of the most recent studies has reported that dried sarcocarp of *C. officinalis* consists of 11 highly polar compounds, particularly, iridoid isomers (7α-O-methylmorroniside, 7β-O-methylmorroniside, 7α-O-ethylmorroniside, and 7β- ethylmorroniside) 26. Gallic acid, 5-hydroxymethylfurfural, morroniside, and loganin are the most abundant compounds in the *C. officinalis* fruit; however, their content could vary with the state of the fruit, depending on whether they are processed or crude. Particularly, loganin possesses immune-regulatory and anti-inflammatory activities, while morroniside is involved in the prevention of diabetic angiopathy 27. In the present study, EECF did not show cytotoxicity at any of the tested concentrations as reported previously 28. A previous study reported that EECF has relatively high DPPH radical scavenging activity, mediated by its antioxidant activity 29. In agreement with previously reported findings, the results of this study indicate that EECF scavenged DPPH radical and superoxide anion (Figures 1B and 1D). It has been reported that EECF contains flavonoids, which are known to possess antioxidant activity via hydrogen donation 28,30. Our results showed that EECF exhibited antioxidant activity by attenuating hydrogen peroxide-induced ROS generation_,_ and ameliorated intracellular ROS generation, as revealed by DCF-DA staining (Figures 1C and 1E).

PM_2.5_-induced ROS caused oxidative damage, which resulted in protein carbonylation, lipid peroxidation, and DNA damage 31. ROS attack proteins by oxidation, which is the main mechanism of protein modification. Furthermore, protein modification can be reversible or irreversible. Protein modification leads to protein carbonylation, protein-protein cross linking, and adduct formation with lipid peroxidation products. Eventually, proteins become fragmented and degraded by ROS-mediated protein modification 32. ROS affect lipids, mainly through hydroxyl radical and hydroperoxyl. Especially, polyunsaturated fatty acids are converted to lipid peroxyl radical and hydroperoxide as the result of oxygen insertion. Eventually, lipid peroxidation negatively affects cellular functions such as protein synthesis and alters biochemical properties 33. It has been reported that PM_2.5_ can arrest the cell cycle, resulting in DNA damage and the level of 8-OHdG (an oxidative DNA adduct) 34. DPPP staining revealed that, EECF has an ability to reduce the PM_2.5_-induced lipid peroxidation (Figure 2A). In addition, our results illustrated that EECF pretreatment significantly attenuated protein carbonyl formation in cells while the comet assay revealed the protective effect of EECF on PM_2.5_-induced DNA damage. EECF significantly attenuated the DNA strand breaking and at 200 µg/mL, also reduced the elevated 8-oxoG level in PM_2.5_-treated cells.

A previous study reported that cellular oxidative stress causes mitochondria stress, which eventually results in cell apoptosis 35. Oxidative stress could be further enhanced by mitochondrial Ca^2+^ accumulation, while the endoplasmic reticulum releases Ca^2+^. As previously reported, ROS degrade the Δψ_m_ 36, and our results revealed that EECF strongly ameliorated PM_2.5_-induced excessive Ca^2+^ accumulation in the cell and mitochondria. This effect restored cellular Ca^2+^ homeostasis and EECF restored the Δψ_m_. In conclusion, our results confirmed that EECF has considerable antioxidant activity against PM_2.5_-induced skin damage.

## References

[1] Hyun YJ, Piao MJ, Kang KA (2019). Effect of fermented fish oil on fine particulate matter-induced skin aging. Mar Drugs.

[2] Li D, Li Y, Li G (2019). Fluorescent reconstitution on deposition of PM2.5 in lung and extrapulmonary organs. Proc Natl Acad Sci USA.

[3] Pan TL, Wang PW, Aljuffali IA (2015). The impact of urban particulate pollution on skin barrier function and the subsequent drug absorption. J Derma Sci.

[4] Rychlik KA, Secrest JR, Lau C (2019). In utero ultrafine particulate matter exposure causes offspring pulmonary immunosuppression. Proc Natl Acad Sci USA.

[5] Park JH, Oh SJ, Lee JH (2019). Effects of Particulate matter on healthy human skin: A panel study using a smartphone application measuring daily skin condition. J Eur Acad Dermatol Venereol.

[6] Patlevič P, Vašková J, Švorc PJ (2016). Reactive oxygen species and antioxidant defense in human gastrointestinal diseases. Integr Med Res.

[7] Hu R, Xie XY, Xu SK (2017). PM2.5 Exposure elicits oxidative stress responses and mitochondrial apoptosis pathway activation in HaCaT keratinocytes. Chin Med J.

[8] Tural S, Koca I (2008). Physico-chemical and antioxidant properties of cornelian cherry fruits (Cornus mas L.) grown in Turkey. Sci Hortic.

[9] Czerwińska ME, Melzig MF (2018). Cornus mas and Cornus officinalis-analogies and differences of two medicinal plants traditionally used. Front Pharmacol.

[10] Huang J, Zhang Y, Dong L (2018). Ethanopharmacoloy, phytochemistry, and pharmacology of Cornus officinalis Sieb. Et Zucc. J Ethnopharmacol.

[11] Bai C, Cao B, Li G (2014). Ecologicle effects on phnotypic, cytological and biochemical diversity of Cornus officinalis germplasm resources in China and USA. Biochem Syst Ecol.

[12] Ma W, Wang KJ, Cheng CS (2014). Bioactiove compounds from Cornus officinalis and their effects on diabetic nephropathy. J Ethnophar.

[13] Ji LL, Wang X, Li JJ (2019). New iridoid derivatives from the fruits of Cornus officinalis and their neuroprotective activities. Molecules.

[14] Jiang J, Chen H, Wang L (2016). Quality evaluation of polar and active component in crude and processed Fructus corni by quantitative analysis of multicomponents with single marker. J Anal Methods Chem.

[15] Piao MJ, Ahn MJ, Kang KA (2018). Particulate matter 2.5 damages skin cells by inducing oxidative stress, subcellular organelle dysfunction, and apoptosis. Arch Toxicol.

[16] Oh MC, Piao MJ, Fernando PM (2016). Baicalein protects human skin cells against ultraviolet B-induced oxidative stress. Biomol Ther.

[17] Piao MJ, Kim KC, Choi JY (2011). Silver nanoparticles down-regulate Nrf2-mediated 8-oxoguanine DNA glycosylase 1 through inactivation of extracellular regulated kinase and protein kinase B in human chang liver cells. Toxicol Lett.

[18] Piao MJ, Kang KA, Zhen AO (2019). Particulate matter 2.5 mediated cutaneous cellular injury by inducing mitochondria-associated endoplasmic reticulum stress: protective effects of ginsenoside Rb1. Antioxidants.

[19] Cha JD, Kim HK, Cha IH (2014). Cytoplasmic HuR expression: correlation with cellular inhibitors of apoptosis protein-2 expression and clinicopathologic factors in oral squamous cell carcinoma cells. Head Neck.

[20] Zhen AX, Piao MJ, Hyun YJ (2019). Diphlorethohydroxycarmalol attenuates fine particulate matter-induced subcellular skin dysfunction. Mar Drugs.

[21] Dryden MS (2009). Skin and soft tissue infection: microbiology and epidemiology. Int J Anti Agents.

[22] Watt SM, Pleat JM (2018). Stem cells, niches and scaffolds: applications to burns and wound care. Adv Drug Deliv Rev.

[23] Sanford JA, Gallo RL (2013). Functions of the skin microbiota in health and disease.

[24] Magnani ND, Muresan XM, Belmonte G (2016). Skin damage mechanisms related to airborne particulate matter exposure. Toxicol Sci.

[25] Park CG, Cho HK, Shin HJ (2018). Comparison of mutagenic activities of various ultra-fine particles. Toxicol Res.

[26] Wang L, Chen H, Jiang Y (2018). Simultaneous determination of 11 high-polarity components from Fructus corni: a quantitative LC-MS/MS method for improved quality control. J Chromatogr Sci.

[27] Cai H, Cao G, Cai B (2013). Rapid simultaneous identification and determination of the multiple compounds in crude Fructus Corni and its processed products by HPLC-MS/MS with multiple reaction monitoring mode. Pharm Biol.

[28] Hwang KA, Hwang YJ, Song J (2016). Antioxidant activities and oxidative stress inhibitory effects of ethanol extracts from Cornus officinalis on raw 264.7 cells. BMC Complement Altern Med.

[29] Lee SE, Hwang HJ, Ha JS (2003). Screening of medicinal plant extracts for antioxidant activity. Life Sci.

[30] Hewage SRM, Piao MJ, Kim KC (2015). Galangin (3,5,7-trihydroxyflavone) shields human keratinocytes from ultraviolet B-induced oxidative stress. Biomol Ther.

[31] Zhen AX, Piao MJ, Hyun YJ (2019). Purpurogallin protects keratinocytes from damage and apoptosis induced by ultraviolet B radiation and particulate matter 2.5. Biomol Ther.

[32] Perluigi M, Di Domenico F, Blarzino C (2010). Effects of UVB-induced oxidative stress on protein expression and specific protein oxidation in normal human epithelial keratinocytes: a proteomic approach. Proteome Sci.

[33] Ayala A, Muñoz MF, Argüelles S (2014). Lipid peroxidation: production, metabolism, and signaling mechanisms of malondialdehyde and 4-hydroxy-2-nonenal. Oxid Med Cell Longev.

[34] Abbas I, Badran G, Verdin A (2019). In vitro evaluation of organic extractable matter from ambient PM2.5 using human bronchial epithelial BEAS-2B cells: Cytotoxicity, oxidative stress, pro-inflammatory response, genotoxicity, and cell cycle deregulation. Environ Res.

[35] Greco V, Longone P, Spalloni A (2019). Crosstalk between oxidative stress and mitochondrial damage: Focus on amyotrophic lateral sclerosis. Adv Exp Med Biol.

[36] Hsu YH, Chuang HC, Lee YH (2019). Traffic-related particulate matter exposure induces nephrotoxicity in vitro and in vivo. Free Radic Biol Med.

